# Supplementary material for: METCAM/MUC18 Plays a Tumor Suppressor Role in the Development of Nasopharyngeal Carcinoma Type I
Source: Int J Mol Sci. 2022 Nov 2;23(21):13389. doi: 10.3390/ijms232113389 (PMC9655335; doi:10.3390/ijms232113389)
Supplement: Supplementary file 1 [file ijms-23-13389-s001.zip › ijms-1967603-supplementary.pdf]

Supplementary materials:

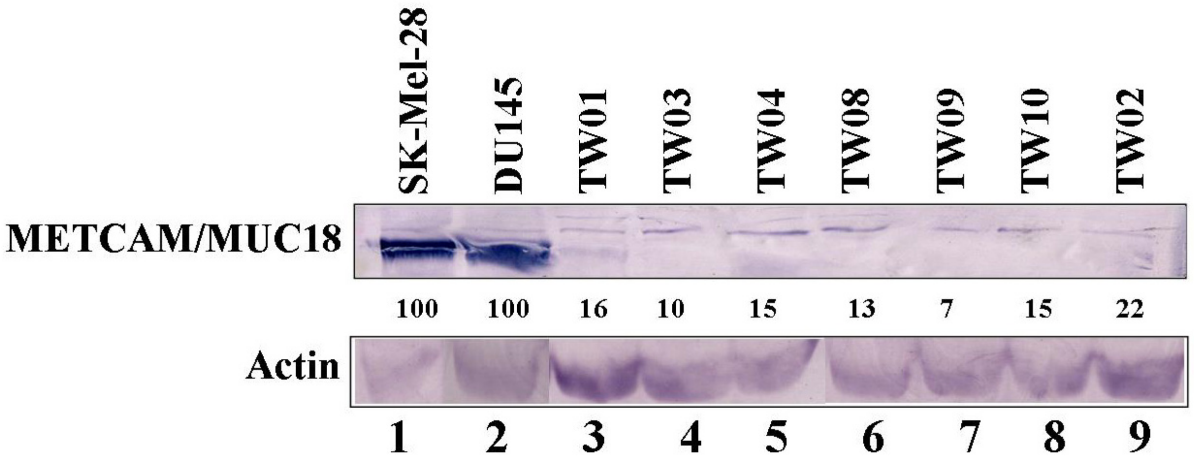

Figure S1. Expression of huMETCAM/MUC18 in seven NPC-TW cell lines. Cultivation of all seven NPC cell lines and WB analysis were carried out as described in "Materials and methods." NPC-TW01, TW02, TW08 and TW10 were established from NPC type I and NPC-TW03, TW04, and TW09 were established from NPC type III [37,38]. The number below each lane was relative expression level of huMETCAM/MUC18 in comparison to that of SK-Mel-28 (assumed as 100%). Actin was the loading control for each lane.
